# Recognition of lameness and decisions to catch for inspection among sheep farmers and specialists in GB

**DOI:** 10.1186/1746-6148-4-41

**Published:** 2008-10-14

**Authors:** J Kaler, LE Green

**Affiliations:** 1 Department of Biological Sciences, University of Warwick, Coventry, CV4 7AL, UK

## Abstract

**Background:**

Epidemiological studies have used farmer estimates of the prevalence of lameness in their flocks. This assumes that farmers can identify lame sheep. Eight movie clips of sheep with locomotion from sound to moderately lame were used to investigate the ability of farmers and sheep specialists to recognise lame sheep. Each participant was asked to complete a form and indicate, for each movie clip, whether they thought the sheep was lame and whether they would catch it if it was the only lame sheep or if 2 – 5, 6 – 10 or > 10 sheep were equally lame. The farmers' responses were compared with their estimates of flock lameness prevalence and the interval between observing a lame sheep and catching it.

**Results:**

178 farmers and 54 sheep specialists participated. Participants could identify even mildly lame sheep but made a separate decision on whether to catch them. This decision was dependent on the severity of lameness and the number of sheep lame in a group. Those who said they would catch the first lame sheep in a group were significantly more likely to catch mildly lame sheep (farmer-reported median prevalence of lameness 5% (IQR: 2%–6%)). In contrast, farmers who waited for several sheep to be lame indicated that they would only catch more severely lame sheep (farmer reported median flock lameness 11% (IQR: 9%–15%)). Approximately 15% of farmers did not catch individual lame sheep (farmer reported median flock lameness 15% (IQR: 10%–15%)). The flock prevalence of lameness increased as time to treatment increased and time to treatment was positively correlated with only catching more severely lame sheep.

**Conclusion:**

If movie-clips are similar to the flock situation, farmers and specialists can recognise even mildly lame sheep but vary in their management from prompt treatment of the first lame sheep in a group to no individual sheep treatments. The former practices would be appropriate to minimise transmission of footrot, a common, infectious cause of lameness and so reduce its incidence. The analysis also suggests that farmers estimate lameness prevalence relatively accurately because farmers who treated the first mildly lame sheep in a group also reported the lowest prevalence of lameness in their flock.

## Background

Farmers notice that sheep in their sheep are lame through visual observation. Previous epidemiological studies that have reported the prevalence of lameness in sheep in the UK [1,2] or prevalence of lameness caused by interdigital dermatitis (ID) and footrot (FR) [2-4] have used farmer opinion and assumed that farmers can identify lame sheep. There is evidence that dairy cow farmers underestimate the proportion of lame cattle in their herds [5]. There are two hypotheses for this under estimation. Either farmers cannot/do not identify lame cows, or farmers only consider a cow lame when it is 'lame enough' (in their opinion) to require treatment. This may also be true for sheep farmers. There is no information on the ability of sheep farmers to identify lame sheep or on their decision to examine a lame sheep. This information is also unknown for sheep advisors.

Footrot, one of the most common cause of lameness, caused by the bacterium *Dichelobacter nodosus*, is infectious [6], and the time to treatment of sheep with FR is associated with the prevalence and incidence of lameness because rapid treatment of a sheep increases its rate of recovery and decreases transmission of *D. nodosus *to other sheep. Previous work has indicated that treatment of sheep lame with FR or ID with parenteral antibacterials led to a decrease in the incidence of FR in the rest of the flock in the following 2 – 4 week period [7] and results from a clinical trial indicated that prompt and frequent (daily – twice weekly) treatment of sheep lame with FR and ID reduced the prevalence and incidence of these conditions [8].

In the current study, a selection of eight movie clips of sheep with a range of locomotion scores was used to investigate farmer and sheep specialist opinion of whether a sheep was lame, whether they would catch and inspect the sheep and how many sheep in a group would need to be equally lame for inspection to occur. The association between the farmer's decision on the number of sheep lame in the group before inspection and the reported time to treatment from first observing a sheep lame was compared with the farmer estimated prevalence of lameness in the flock.

## Methods

### Study population

Data were collected from farmers at three farmer events conducted by the English Beef and Lamb Executive (EBLEX) in Devon (n = 73), Newark (n = 86) and Norfolk (n = 33) in 2007. These events were advertised by EBLEX as a part of their Better Returns Programme  and were focussed on lameness management and worm control. In addition, data were also collected from the Sheep Veterinary Society (SVS) meeting in September 2007 in Devon, England.

### Study design

Before a talk and discussion on lameness, farmers were asked to complete a brief questionnaire with questions pertaining to their involvement in the day to day management of sheep, the number of breeding ewes, the average percent of ewes lame in their flock in 2006 and their usual time to treatment from observation of a lame sheep. Delegates at the SVS meeting were asked about their profession and whether they personally had a care of a flock of sheep. All participants were then asked to complete eight identical questions (Table 1) as they watched eight movie clips of sheep with locomotion scores 0 (n = 1), 1 (n = 1), 2 (n = 1), 3 (n = 4) and 4 (n = 1) (Table 2) [9]. The participants did not discuss their answers and were not told the severity of the locomotion score of the sheep, which were not ordered.

**Table 1:** Question asked to participants for each movie clip

Question	Clip X
1	Do you think this sheep is lame? *(please tick one box)*
	□ Yes □ No □ Don't know
	*If *Yes or Don't know, *please go to Question 2, if *No *please wait for next clip*
	
2	Would you catch this sheep with intention to treat? (*please tick*)
	Would you catch this sheep with intention to treat? (*please tick*)
	□ Yes, always, even if this is the first lame sheep in the group
	□ Yes, when at least
	□ 2 – 5 of the sheep in the group are lame
	□ 6 – 10 of the sheep in the group are lame
	□ 11 – 20 of the sheep in the group are lame
	□ more than 20 of the sheep in the group are lame
	□ Don't know

**Table 2:** Locomotion scoring system (source Kaler et al., in press)

**Scale**	**0**	**1**	**2**	**3**	**4**	**5**	**6**
**Posture and locomotion**							
Bears weight evenly on all four feet	✓						
Uneven posture, but no clear shortening of stride		✓	✓	✓	✓	✓	
Short stride on one leg compared with others		✓	✓	✓	✓	✓	
Visible nodding of head in time with short stride			✓				
Excessive flicking of head, more than nodding, in time with short stride				✓	✓	✓	
Not weight bearing on affected limb when standing				✓	✓	✓	
Discomfort when moving				✓	✓	✓	
Not weight bearing on affected limb when moving					✓	✓	
Extreme difficulty rising						✓	
Reluctant to move once standing						✓	
More than one limb affected						✓	
Will not stand or move							✓

Descriptions of the locomotion scores and the method for making the movie clips of sheep are described in [9]. Each movie clip was 20 – 22 second long and was played once within a Microsoft Power Point presentation. The questionnaire took approximately 10 minutes to complete. Data were entered in Microsoft Access 2000 (Microsoft) and checked for errors. The analyses were carried out in Stata 9 SE (Statacorp, USA).

### Statistical analysis

All the farmers and sheep specialists who were involved in the day to day management of sheep were included in the analysis. Comparisons were made between farmers from different groups with respect to the median percent of lame ewes in 2006, the median number of breeding ewes and the time to treatment of lame sheep. In addition, the responses of the participants on identification of lame sheep and their decision to catch them by each locomotion score were compared. Proportions were compared with χ^2 ^test and medians with Kruskal-Wallis test [10] with significance at p ≤ 0.05. Where the categories had low response counts, data were merged with the closest neighbouring category for statistical analysis.

The participating farmers were grouped into four categories: a) farmers who caught the first lame sheep with locomotion scores ≥2 b) farmers who caught the first lame sheep with locomotion scores ≥3 c) farmers who caught first lame sheep with locomotion score = 4 only d) farmers who did not catch the first lame sheep. The significant differences in rank distributions of farmer estimated prevalence of lameness and number of breeding ewes by category was determined by a nonparametric test for trend, an extension of the Wilcoxon rank sum test [11]. A similar test was used to compare estimates of prevalence of lameness by reported time to treatment of lame sheep from first observing them lame and decisions on the minimum number of sheep that were lame in the group (e.g. 1, 2 – 5, 6 – 10 and > 10) before farmers caught sheep with locomotion scores 2, 3 or 4.

## Results

### Background information

A total of 94% (69/73) of farmers from Devon, 93% (80/86) farmers from Newark and 88% (29/33) of farmers from Norfolk who attended the events were involved in day to day management of sheep. Farmers from Devon, Newark and Norfolk had a median number of breeding ewes of 150 (inter-quartile range (IQR): 60 – 350), 200 (IQR: 100 – 320) and 120 (IQR: 50 – 220) respectively. The farmer reported median prevalence of lameness in ewes in 2006 in these same three groups was 8 (IQR: 6 – 10), 5 (IQR: 3 – 10) and 5 (IQR: 3 – 10) respectively. There was no significant difference between the groups of farmers with respect to the median number of breeding ewes (p > 0.05) or the median prevalence of lameness (p > 0.05).

The average percentage of farmers who treated sheep on the first day or within three days of observing them lame was 18% and 38% respectively. Thirty percent of farmers treated sheep within a week of seeing them lame and 14% farmers did not catch individual sheep at all and only treated lame sheep at routine gatherings. There were no significant differences between the groups of farmers from the three regions with respect to the time to treatment of lame sheep (p > 0.05). Out of 54 delegates who completed the questionnaire at the Sheep Veterinary Society meeting; 35 were veterinarians and 19 were specialist advisors. Twenty-one delegates (15 veterinarians) had care of a flock of sheep.

### Identification of lame sheep by locomotion score

**Figure 1:**
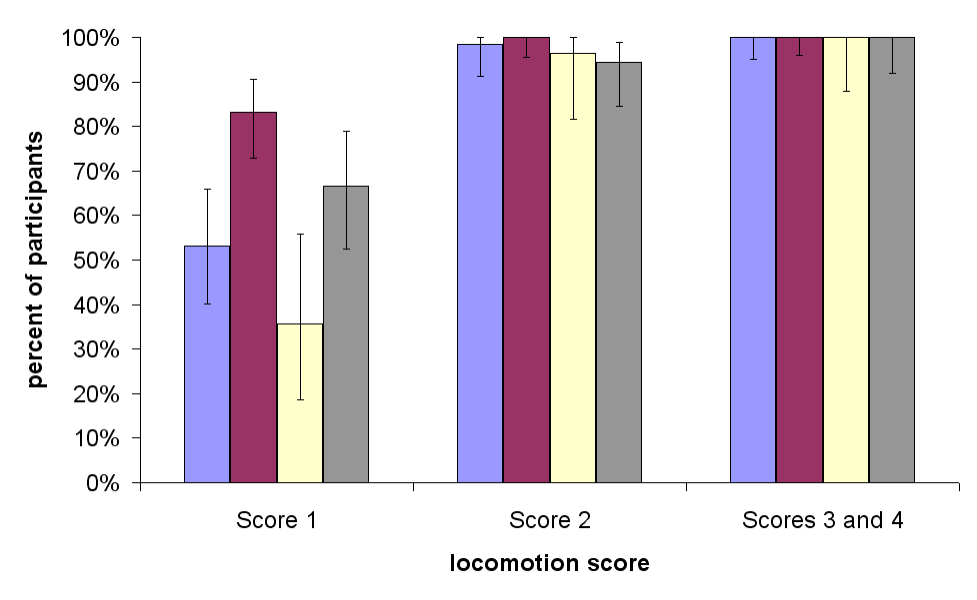
**Percentage of participants by group who considered sheep with locomotion scores 1, 2 and 3 or 4 were lame**. *Key: blue = Devon, red = Newark, yellow = Norfolk, grey = Sheep Veterinary Society.

Approximately 79% (48/61), 91% (73/80), 75% (21/28) and 77% (41/53) of participants from Devon, Newark, Norfolk and SVS respectively (p > 0.05), considered that the sheep with locomotion score 0 was sound (not lame). A significantly (p < 0.05) higher (83%) percentage of farmers from Newark considered that the sheep in the movie clip with locomotion score 1 was lame, compared with 53% (33/62), 36% (10/28) and 67% (36/54) participants from Devon, Norfolk and SVS respectively (Figure 1). Over 90% of the participants considered that the sheep with locomotion score 2 was lame; and all the participants considered that the sheep with locomotion score 3 or 4 were lame (Figure 1).

### Catching lame sheep by locomotion score

**Figure 2:**
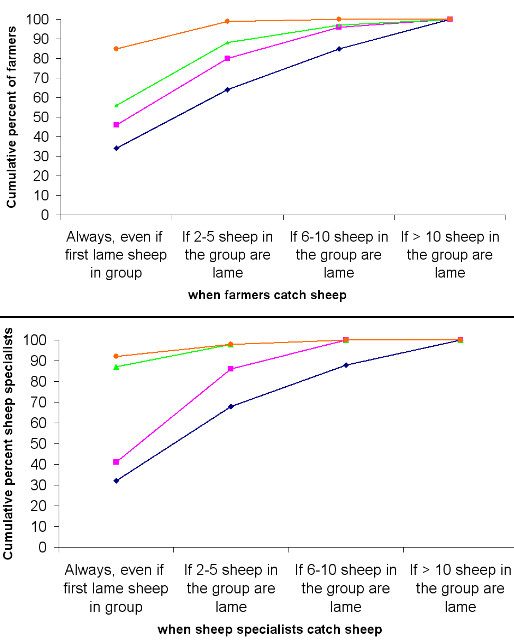
**Cumulative percentage of farmers and sheep specialists by decision to catch sheep with locomotion scores 1, 2, 3 or 4**. *Key – blue = Locomotion score 1, pink = Locomotion score 2, green = Locomotion score 3 (median percent of the four clips is presented), orange = Locomotion score 4.

Overall there was no significant difference (p > 0.05) between groups of farmers with respect to their decisions to catch lame sheep by locomotion score. Only 11 farmers out of 28 who considered that the sheep with locomotion score 0 was lame, said that they would catch it; 2 reported that they would catch the first lame sheep, 2 when at least 2 – 5 sheep in the group were lame, 3 when > 10 sheep in the group were lame and 4 did not know. There was a significant increase in the percentage of farmers and sheep specialists who would catch the first lame sheep as the locomotion score increased from 1 to 4 (p < 0.05) (Table 3, Figure 2). A similar percentage of farmers (34%) and sheep specialists (32%) reported that they would catch the first lame sheep in a group with locomotion score 1; approximately 46% (38% – 55%) of farmers and 41% (27% – 56%) of sheep specialists reported that they would catch the first lame sheep with locomotion score 2 (Table 3) (p > 0.05). In addition, all the participants who said that they would catch the first lame sheep with locomotion scores of 1 or 2, would have caught the first lame sheep in a group with locomotion scores 3 and 4.

**Table 3:** Farmer and sheep specialist decisions to catch lame sheep by locomotion scores 1, 2, 3 or 4

Locomotion score	When catch lame sheep			
	
	First lame sheep in the group (%, 95% CI)	2–5 sheep in the group lame (%, 95% CI)	2–5 sheep in the group lame (%, 95% CI)	> 10 sheep in the group lame (%, 95% CI)
Score 1				
Farmers (N = 100)	34 (25 – 44)	30 (21 – 40)	21 (14 – 30)	15 (9 – 24)
Sheep specialists (N = 34)	31 (17 – 49)	34 (19 – 52)	20 (8 – 37)	12 (3 – 27)
Score 2				
Farmers (N = 142)	46 (38 – 55)	33 (25 – 41)	17 (11 – 24)	4 (1 – 8)
Sheep specialists (N = 49)	41 (27 – 56)	45 (31 – 60)	14 (6 – 27)	
Score 3				
Farmers (N = 161)	56 (48 – 64)	56 (48 – 64)	56 (48 – 64)	56 (48 – 64)
Sheep specialists (N = 53)	87 (85 – 90)	11 (9 – 13)	2 (1 – 4)	
Score 4				
Farmers (N = 164)	85 (79 – 90)	14 (9–20)	1 (0 – 3)	
Sheep specialists (N = 53)	92 (82 – 98)	6 (1 – 16)	2 (0 – 10)	

The overall average median percentage of farmers that would have caught the first lame sheep with locomotion score 3 in the four movie clips was 56% (48% – 64%). A significantly (p < 0.05) higher percent of delegates at SVS (87%; 95% CI: 85% – 90%) reported that they would have caught the first lame sheep with locomotion score 3. The majority of farmers (85% (79% – 90%)) and sheep specialists (92% (82% – 98%)) said that they would catch the first lame sheep with locomotion score 4 (Table 3). There was no significant difference between farmers and sheep specialists in their responses on decisions to catch sheep with locomotion score 4 (p > 0.05) (Table 3).

As the locomotion score increased, the number of sheep lame in the group before participants' inspected the sheep decreased (Figure 2, Table 3).

### Associations between farmer estimated prevalence of lameness, number of breeding ewes and when they would catch lame sheep

There were no significant differences in the median number of ewes per farm by catching behaviour (p_trend _> 0.05, Table 4). However, the median farmer estimated prevalence of lameness in ewes was lower as farmers reported that they would catch the first lame sheep with a low locomotion score (p_trend _< 0.05, Table 4).

**Table 4:** Median prevalence of lameness and number of breeding ewes by decisions to catch the first lame sheep by locomotion score. IQR = interquartile range

Decisions to catch	Median lameness (IQR)	N	Median number of breeding ewes (IQR)	N
Would catch the first lame sheep with locomotion scores ≥ 2	5 (2 – 6)	59	150 (50 – 320)	67
Would catch the first lame sheep with locomotion score ≥ 3	10 (5 – 10)	40	155 (80 – 350)	41
Would catch the first lame sheep with locomotion score 4 only	10 (8 – 10)	8	200 (150 – 400)	10
Would never catch the first lame sheep	11 (9 – 15)	24	200 (120 – 300)	27

P trend*		< 0.05		> 0.05

**Figure 3:**
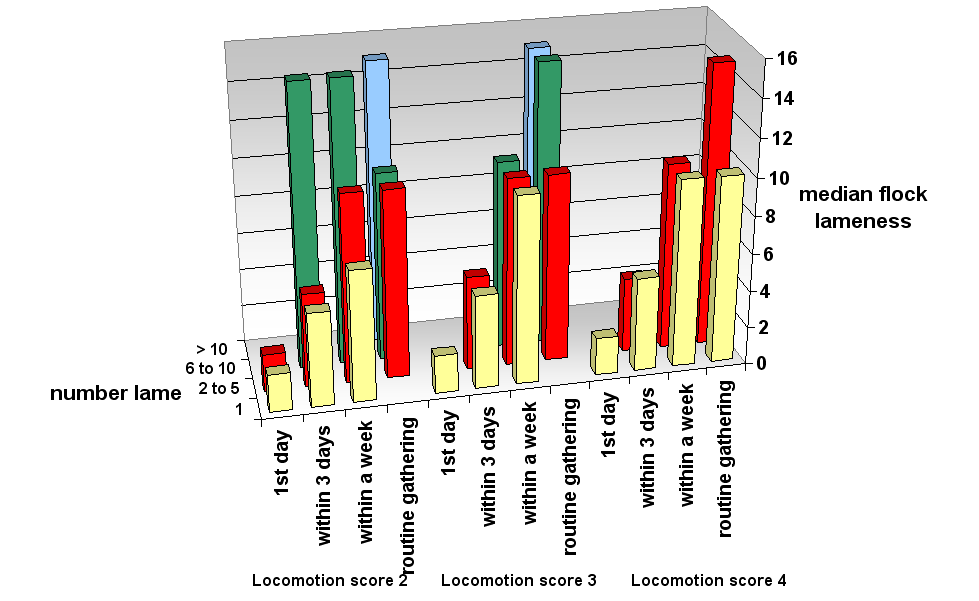
Time to treatment and decisions to catch lame sheep of locomotion scores 2, 3 or 4 by estimated flock lameness * Key: Yellow – catch the first lame sheep, Red – catch when 2 to 5 sheep in the group are lame, Green – catch when 6 – 10 sheep in the group are lame, Blue – catch when > 10 sheep in the group are lame.

The median prevalence of lameness by decisions to catch sheep by locomotion score is presented in Table 5 (with a graphical presentation of trends in Figure 3), together with the reported time from observation to treatment.

**Table 5:** Farmer estimated prevalence of lameness by time to treatment and decisions to catch lame sheep IQR = interquartile range

Locomotion Score	Time to treatment	Farmer decision to catch lame sheep									
		
		First lame sheep in group		2 – 5 lame sheep in the group		6 – 10 lame sheep in the group		> 10 lame sheep in the group		*Total*	
		Median lameness (IQR)	N	Median lameness (IQR)	N	Median lameness (IQR)	N	Median lameness (IQR)	N	Median lameness (IQR)	N
Score 2	Treat first day	2 (1 – 5)	21	3 (1 – 6)	2					2 (1–5)	23
	Treat ≤ 3 days	5 (2 – 7)	27	5 (4 – 10)	18	15 (5 – 20)	3			5 (3–10)	48
	Treat ≤ 7 days	7 (4 – 15)	8	10 (10 – 15)	20	15 (10 – 20)	9	15 (15 – 15)	1	10 (8–15)	38
	Treat only at routine gatherings			10 (8 – 20)	7	10 (6 – 15)	7	15 (15 – 20)	5	15 (10–15)	19
	*Total*	5 (2–6)	56	10 (5–10)	47	10 (10–15)	19	15 (15–23)	6		
Score 3	Treat first day	2 (1 – 6)	25							2 (1–6)	25
	Treat ≤ 3 days	5 (5 – 8)	33	5 (5 – 10)	16	15 (15 – 15)	1			5 (3–10)	50
	Treat ≤ 7 days	10 (5 – 15)	13	10 (8 – 15)	27	10 (10 – 15)	3			10 (5–15)	43
	Treat only at routine gatherings	6 (6 – 6)	1	10 (8 – 15)	8	15 (10 – 15)	9	15 (15 – 23)	3	15 (10–15)	21
	*Total*	5 (2–10)	72	10 (5–15)	51	15 (10–15)	13	15 (15–23)	3		
Score 4	Treat first day	2 (1 – 5)	26							2 (1–5)	26
	Treat ≤ 3 days	5 (4 – 10)	50	4 (4 – 15)	3					5 (4–10)	53
	Treat ≤ 7 days	10 (5 – 15)	29	10 (8 – 15)	13					10 (5–15)	42
	Treat only at routine gatherings	10 (10 – 10)	10	15 (15 – 20)	9					15 (10–15)	19
	*Total*	5 (3–10)	115	15 (8–15)	25						

The results from only one of the clips with locomotion score 3 is included in Table 5 (the trend was similar for all four clips of locomotion score 3).

There was a significant association between farmer reported time to treatment and the minimum number of lame sheep in a group before sheep with locomotion score 2, 3 or 4 were caught (p < 0.05) (Table 5, Figure 3). Almost all the farmers who reported that they would treat lame sheep of any severity the first day that they saw them lame also always caught the first lame sheep in a group. Similarly, the majority (> 93%) of farmers, who reported that they treated lame sheep within 3 days of observing them lame, either always caught the first lame sheep or the first 2 – 5 lame sheep in the group. However, the majority of the farmers who treated lame sheep within a week or only at routine gatherings, reported that they would not catch the first lame sheep in the group until they saw the sheep with locomotion score 4 (Table 5).

There was a significant increase in the median farmer reported prevalence of lameness with increased time from observation to treatment of lame sheep (p_trend _< 0.05). The median prevalence of lameness also increased as the minimum number of sheep lame in the group increased (p_trend _< 0.05) (Table 5).

## Discussion

A key finding from this study is that farmers and sheep specialists recognised even mildly lame sheep. However, whilst they considered that a sheep with a locomotion score as low as 2 was lame, a proportion of flock carers did not catch an individual lame sheep until the locomotion score was 4 (when a sheep is not bearing weight on its affected limb while moving and standing). One limitation of this study is that we did not ask how farmers observe sheep to detect lameness. Sheep are a prey animal and will mask mild disease if they consider that they are in a threatening situation. Consequently, good observation of lameness, especially mildly lame sheep, is more accurate when sheep are undisturbed. Despite this he farmer estimates of prevalence of lameness seem likely to be accurate, because their management (waiting until several sheep were lame, or only treating lame sheep at routine gatherings) is consistent with a higher prevalence of lameness. Leaving lame sheep in the flock untreated will increase the prevalence of lameness unless spontaneous recovery occurs. One concern is that the flock prevalence of lameness might be either the percentage of lame sheep or the percentage of sheep that they would catch. However, given that farmers who would have caught more severely lame sheep only when a number of them were lame also reported the highest prevalence of lameness, it would appear that farmer self reporting of prevalence of lameness is reasonably accurate.

The high correlation between the time to treatment and farmer decisions to always catch the first lame sheep with varying locomotion suggests that the information collected in the questionnaire on farmer decisions is reliable. Variation in decisions to catch may be a result of an inherent assumption among farmers and sheep specialists that sheep with lower locomotion scores do not have foot lesions, are not in pain, will recover without treatment or will become more lame and then require treatment. Farmers attribute the majority of their flock lameness to two foot lesions; ID (caused by *F. necrophorum*) and FR [2], and, even mildly lame sheep have these lesions [12]. Thus, considering this evidence and the fact that footrot is infectious [6], not catching mildly lame sheep, increasing the time to treatment from first observing a lame sheep, or waiting until a certain number in a group are lame would allow *D. nodosus *to spread and hence increase the incidence of FR and therefore of lameness if the affected sheep had footrot or interdigital dermatitis. The findings from this study support this hypothesis because farmers who reported that they always caught the first lame sheep in a group with a low severity of locomotion and treated lame sheep within 1 or 3 days of seeing them lame had a significantly lower median flock lameness than farmers who did not practice this management. It is likely that these farmers reduced transmission of infectious causes of lameness and aided recovery of sheep with other, non-infectious causes of lameness. In contrast, the farmers who treated sheep at routine gatherings only had the highest median prevalence of lameness which would be because of delayed treatment of non-infectious and infectious causes of lameness.

There was a good percentage raw agreement (> 74%) between participants responses to whether sheep of locomotion scores 2, 3 and 4 were lame. However, there was disagreement about whether the sheep with locomotion scores 1 and zero were lame. This was also reported in [9] where disagreement between observers trained to locomotion score sheep was greatest for scores 0 and 1 when 30 movie clips were used to estimate reliability of locomotion score as a useful research tool. This suggests that differentiating sound from very mildly lame sheep is more difficult, but once sheep are locomotion score 2 or above (defined in Table 2) the majority of participants recognise lame sheep. In addition, their decision to catch sheep varied consistently by the severity of the locomotion score (Table 3). Both these facts indicate that farmers and sheep specialists were differentiating between different severities of locomotion although not asked this directly. However, the results of the current study suggest that we should not be using locomotion scoring as a system of management of lame sheep but encouraging farmers and advisors to control lameness through examination of all lame sheep, whatever the severity of lameness.

There may be several factors that influence a farmer's decision to catch lame sheep. Although one might expect farmers with small flocks to be more likely to catch individual lame sheep with lower locomotion scores, there was no significance difference in the median number of breeding ewes on the farms where farmers always caught the first lame sheep with low locomotion score and those where farmers did not. The type of handling facilities and the amount of labour and time available might also affect a farmer's decision to catch lame sheep. With portable handling facilities and more labour and time available it may be that farmers are more able to always catch the first lame sheep even those with low locomotion score. Information on use of these facilities was not collected in this study.

Various considerations and assumptions were made before deciding on the number of movie clips used in this study. There was a time constraint, all the farmer meetings and SVS meeting had a specific agenda and participants attended those meeting with a purpose so we wished to minimise the time that the quiz took. Clips of locomotion scores 5 and 6 (Table 2) were not included in the study because it was assumed that most farmers would catch these very lame sheep and so there would not be any variability in the results. This was a reasonable assumption given the uniform response to the sheep with locomotion score 4, which all participants considered lame and would catch. Four clips of sheep with score 3 were included because locomotion score 3 is the middle of the locomotion scoring scale and, based on personal observation, it was assumed that farmers do not always catch sheep with locomotion score 2 but do catch sheep with locomotion score 4 so most variability would occur at score 3. There was variation in farmers decisions to catch between scores but also within the sheep with locomotion score 3. Although the locomotion score is categorical, there is a continuous range of abnormalities within any score, and one of the four sheep in this category had excessive flicking of head compared with the other three sheep: farmers and sheep specialists were more likely to state that they would catch this sheep than the other three.

Although there were no significant differences between the three groups of farmers with respect to the overall results of the study, the sample of farmers was not random. Similarly, the sample of sheep specialists was a convenience sample from a SVS meeting. This might impact on the estimates of the percent of sheep caught by severity and time and number lame before inspection, but the associations between low estimated prevalence of lameness and rapid treatment of the first mildly lame sheep in the group is likely to remain.

The use of movie clips of sheep with varying locomotion was a useful approach to gather information on farmer and specialist decisions to catch lame sheep because it provided identical observations for all participants. At a farm visit it might not be possible to see a range of severities of locomotion at one time and it is possible that farmers' responses might change for sheep in their own flock. In addition, farmers and specialists were probably less intimidated to report to their decisions individually on paper than they might be in a face to face situation on farm. Evidence suggests that respondents respond differently to behavioural or attitudinal questions in self administered questionnaires and face to face interviews; they feel more pressurised to give a 'socially desirable' answer in face to face interviews [13].

## Conclusion

Assuming that recognition of lameness from movie clips is similar to recognition of lameness in live sheep, farmers can recognise even mildly lame sheep but make a separate decision on when to catch a sheep for inspection. In addition, the prevalence of lameness was higher in flocks where farmers either waited longer than 3 days to treat a lame sheep, or waited until a certain number of sheep in the group were lame before inspection or both. The current study indicates that farmers have the skills to follow the advice from previous research to minimise lameness in sheep; they inspect the first mildly lame sheep in a group within 1 – 3 days of it first being lame.

## Authors' contributions

JK participated in the design of the study and data collection, performed the data analysis and drafted the manuscript. LEG participated in the design of the study and data collection and advised on data analysis and assisted with writing the manuscript.

## References

[B1] Grogono-Thomas R, Wilsmore AJ, Simon AJ, Izzard KA (1994). The use of long-acting oxytetracycline for the treatment of ovine footrot. British Veterinary Journal.

[B2] Kaler J, Green LE (2008). Naming and recognition of six foot lesions of sheep using written and pictorial information: A study of 809 English sheep farmers. Preventive Veterinary Medicine.

[B3] Wassink GJ, Grogono-Thomas R, Moore LJ, Green LE (2003). Risk factors associated with the prevalence of footrot in sheep from 1999 to 2000. Veterinary Record.

[B4] Wassink GJ, Grogono-Thomas R, Moore LJ, Green LE (2004). Risk factors associated with the prevalence of interdigital dermatitis in sheep from 1999 to 2000. Veterinary Record.

[B5] Whay HR, Main DCJ, Green LE, Webster AJF (2003). Assessment of the welfare of dairy cattle using animal-based measurements: Direct observations and investigation of farm records. Veterinary Record.

[B6] Beveridge WIB (1941). Footrot in sheep: A transmissible disease due to infection with Fusiformis nodosus. Studies on its cause, epidemiology and control. Council for Scientific and Industrial Research, Bulletin No 140.

[B7] Green LE, Wassink GJ, Grogono-Thomas R, Moore LJ, Medley GF (2007). Looking after the individual to reduce disease in the flock: A binomial mixed effects model investigating the impact of individual sheep management of footrot and interdigital dermatitis in a prospective longitudinal study on one farm. Preventive Veterinary Medicine.

[B8] Hawker EM (2007). An Intervention study to minimise footot in sheep. MSc thesis.

[B9] Kaler J, Wassink GJ, Green LE (2008). The inter- and intra-observer reliability of a locomotion scoring scale for sheep. The Veterinary Journal.

[B10] Petrie A, Watson P (1999). Statistics for veterinary science and animal science. Blackwell Science.

[B11] Jack C (1985). A wilcoxon-type test for trend. Statistics in Medicine.

[B12] Kaler J (2008). Epidemiological investigations into lameness in sheep. PhD thesis.

[B13] Krysan M, Schuman H, Scott LJ, Beatty P (1994). Response rates and response content in mail surveys versus face-to-face surveys. Public Opinion Quarterly.

